# Blood Pressure Regulation by the Carotid Sinus Nerve: Clinical Implications for Carotid Body Neuromodulation

**DOI:** 10.3389/fnins.2021.725751

**Published:** 2022-01-10

**Authors:** Silvia V. Conde, Joana F. Sacramento, Bernardete F. Melo, Rui Fonseca-Pinto, Mario I. Romero-Ortega, Maria P. Guarino

**Affiliations:** ^1^Faculdade de Ciências Médicas, Chronic Disease Research Center (CEDOC), NOVA Medical School, Universidade NOVA de Lisboa, Lisbon, Portugal; ^2^ciTechCare, School of Health Sciences, Polytechnic of Leiria, Leiria, Portugal; ^3^Department of Biomedical Engineering, University of Houston, Houston, TX, United States

**Keywords:** carotid body, carotid sinus nerve, hypoxia, blood pressure, neuromodulation

## Abstract

Chronic carotid sinus nerve (CSN) electrical modulation through kilohertz frequency alternating current improves metabolic control in rat models of type 2 diabetes, underpinning the potential of bioelectronic modulation of the CSN as a therapeutic modality for metabolic diseases in humans. The CSN carries sensory information from the carotid bodies, peripheral chemoreceptor organs that respond to changes in blood biochemical modifications such as hypoxia, hypercapnia, acidosis, and hyperinsulinemia. In addition, the CSN also delivers information from carotid sinus baroreceptors—mechanoreceptor sensory neurons directly involved in the control of blood pressure—to the central nervous system. The interaction between these powerful reflex systems—chemoreflex and baroreflex—whose sensory receptors are in anatomical proximity, may be regarded as a drawback to the development of selective bioelectronic tools to modulate the CSN. Herein we aimed to disclose CSN influence on cardiovascular regulation, particularly under hypoxic conditions, and we tested the hypothesis that neuromodulation of the CSN, either by electrical stimuli or surgical means, does not significantly impact blood pressure. Experiments were performed in Wistar rats aged 10–12 weeks. No significant effects of acute hypoxia were observed in systolic or diastolic blood pressure or heart rate although there was a significant activation of the cardiac sympathetic nervous system. We conclude that chemoreceptor activation by hypoxia leads to an expected increase in sympathetic activity accompanied by compensatory regional mechanisms that assure blood flow to regional beds and maintenance of hemodynamic homeostasis. Upon surgical denervation or electrical block of the CSN, the increase in cardiac sympathetic nervous system activity in response to hypoxia was lost, and there were no significant changes in blood pressure in comparison to control animals. We conclude that the responses to hypoxia and vasomotor control short-term regulation of blood pressure are dissociated in terms of hypoxic response but integrated to generate an effector response to a given change in arterial pressure.

## Introduction

Carotid sinus nerve (CSN) denervation improves glucose homeostasis in insulin-resistant and glucose-intolerant rats (Ribeiro et al., 2013; Sacramento et al., 2017, 2018). Electrical modulation of the CSN through kilohertz frequency alternating current is also shown to revert dysmetabolism metabolic in animal models of type 2 diabetes (Sacramento et al., 2018). In the context of an innovative therapeutic approach, termed bioelectronic medicines, in which individual nerve fibers are targeted in pathological conditions to restore functionality, the CSN emerges with a vast therapeutic potential in cardiometabolic disorders (Sacramento et al., 2018; Conde et al., 2020). Still, the positive effects of CSN blockade may be hindered by adverse effects associated with permanent loss of function (Conde, 2018). The CSN carries sensory information from the carotid bodies (CB), peripheral chemoreceptor organs that respond to changes in blood biochemical modifications such as hypoxia, hypercapnia, acidosis, and hyperinsulinemia (Gonzalez et al., 1994; Conde et al., 2014). In addition, the CSN also delivers information from carotid sinus baroreceptors, mechanoreceptor sensory neurons directly involved in the control of blood pressure (Marshall, 1998; Chapleau et al., 2001). The CB is implicated in the pathophysiology of several cardiovascular diseases, such as chronic heart failure (Del Rio et al., 2013; Schultz et al., 2013) and several forms of hypertension (Prabhakar and Peng, 2004; Abdala et al., 2012; Paton et al., 2013) playing a fundamental role in the genesis and maintenance of these diseases. It is shown that CSN inputs from the CB contribute to the elevated systemic sympathetic tone being critical for the genesis and maintenance of hypertension in spontaneously hypertensive rats (Abdala et al., 2012; McBryde et al., 2013). Also, it is shown that rats with chronic heart failure develop increased CB chemoreflex drive and chronic central presympathetic neuronal activation, increased sympathetic outflow, increased breathing variability, and apnea incidence as well as desensitization of the baroreflex, these effects being reduced by CB ablation (Del Rio et al., 2013). Together, these results confirm the role of the CB in regulating blood pressure and cardiac performance via sympathetic nervous system (SNS) activation. The interaction between these powerful reflex systems—chemoreflex and baroreflex—whose sensory receptors are in anatomical proximity, might represent a shortcoming to the development of selective bioelectronic tools to modulate the CSN.

The chemoreflex and baroreflex control the cardiovascular system via the profound influences they exert on autonomic outflow (Marshall, 1994). Disclosing the interdependency between these two reflex responses, particularly in the presence of stimuli such as hypoxia or ischemia (Marshall, 1998), is required to address pathophysiological mechanisms and therapeutics in cardiometabolic diseases. Although chemoreceptors are known for their role in the control of ventilation, they also modulate cardiovascular, endocrine, and renal systems. In contrast with the respiratory responses, the cardiovascular responses to CB stimulation are surrounded by a lot of controversies. They were extensively studied in the 70’s and 80’s although, as pointed out by Marshall (1994), multiple confounding factors contribute to an uneven interpretation of results, such as hyperventilation, hypocapnia, pulmonary stretch/vagal activation, central respiratory drive, baroreceptor involvement, circulating catecholamines, and the preparation studied (specie, awake/anesthetized animal, among others). The baroreceptors, on the other hand, are mechanoreceptor sensory neurons that respond to mechanical deformation of the nerve endings during distension of the arterial wall (Chapleau et al., 2001). They provide information to the solitary nucleus in the medulla oblongata to influence cardiac output and systemic vascular resistance through a negative feedback system called the baroreflex. Baroreceptor activation induces hypotension, and baroreceptor resection results in systemic hypertension (Irigoyen et al., 1991; Kougias et al., 2010). Baroreflex sensitivity is decreased in numerous pathological states, including chronic arterial hypertension, heart failure, obesity, and diabetes mellitus (Chapleau et al., 2001; Limberg et al., 2015). There is still debate in the literature regarding the interdependency between these two reflex responses, particularly in the presence of stimuli such as hypoxia.

Our group is dedicated to understanding the physiology of the CB chemoreceptors and to look at the CSN as a bioelectronic medicine target. Herein, we designed experiments to explore the crosstalk between CB-mediated blood pressure responses using different stimuli to the CSN from hypoxic hypoxia to ischemic hypoxia and electrical stimulation or high-frequency blocking. The primary objective of the study is to determine if electrical neuromodulation of CSN affects systemic blood pressure and cardiac autonomic function in both normoxic and hypoxic conditions.

## Materials and Methods

### Animals

All animal experimental and care procedures were approved by the Ethics Committee and by the Animal Welfare Body of Faculdade de Ciências Médicas| Nova Medical School and by the Direcção Geral de Veterinária, Portugal. Principles of laboratory care were followed following the European Union Directive for Protection of Vertebrates Used for Experimental and Other Scientific Ends (2010/63/EU). Experiments were performed in male Wistar Han rats (220–260 g) at 10–12 weeks old obtained from the vivarium of the Faculdade de Ciências Médicas, Universidade Nova de Lisboa, Lisboa, Portugal. Animals were kept under controlled temperature and humidity (21 ± 1°C; 55 ± 10% humidity) with a 12 h light/dark cycle and *ad libitum* access to food and water. In protocols requiring anesthesia, rats were anesthetized with sodium pentobarbital (60 mg kg^–1^ i.p.) and supplemented intravenously with 10% of the initial dose as necessary to make them areflexic to a nociceptive stimulus (effects of corneal reflexes and pinch to the front paw on the rise in arterial blood pressure). Body temperature was maintained close to 37 ± 1°C using a heated underblanket controlled by a rectal thermistor probe.

### Evaluation of Blood Pressure and Heart Rate Responses to Acute Hypoxia in Conscious and Anesthetized Animals

To perform the set of experiments in conscious animals, rats were implanted with telemetry devices for blood pressure and heart rate (HR) continuous recording (HD-S10, Data Sciences Corporation, United States) under ketamine [75 mg/kg body weight (i.p.), Nimatek, Dechra, Northwich, United Kingdom]/metedomidine [0.5 mg/kg body weight (i.p.), Sedator^®^, Dechra, Northwich, United Kingdom] anesthesia and buprenorphine (10 μg/kg, Bupaq^®^, Richter Pharma AG, Wels, Austria) analgesia. In brief, the abdominal aorta was exposed via a ventral midline incision in the abdominal cavity, and the radio telemetry transmitter was implanted aseptically and sealed with a drop of tissue adhesive (Vetbound, 3M Company, St. Paul, MN, United States). The body of the transmitter was then placed on top of the intestines and secured to the abdominal muscle. Anesthesia was reversed with atipamezole [0.25 mg/kg in 2 ml (i.p.), Antisedan^®^, Zoetis, New Jersey, United States]. Animals were treated for 3 days with the non-steroidal anti-inflammatory drug carprofen [5 mg/kg/ml (s.c.), Rimadyl™, Pfizer, New York, United States] and allowed to recover from surgery for 15 days. Blood pressure, HR, and autonomic responses to hypoxic hypoxia were evaluated in conscious animals implanted with telemeters by placing the animals in a polypropylene cage equipped with gas injectors and sensors for oxygen (O_2_). The hypoxic hypoxia protocol consisted of submitting the animals to 30 min baseline recording at normoxia (20% O_2_ balanced N_2_) followed by 15 min hypoxia (10% O_2_ balanced N_2_), followed by 10 min normoxia. Systolic (SBP), diastolic (DBP), and mean blood pressure (MBP) and HR were obtained via radio frequency signals through the Data Acquisition System from Data Sciences International (DSI, St. Paul, MN). Blood pressure and HR measurements were obtained during 1-s sampling periods and averaged. For measurement of blood pressure and HR in an anesthetized setting, animals were administered sodium pentobarbital (60 mg kg-1 i.p.), and the femoral artery and vein were catheterized under a dissection microscope. The femoral artery catheter was connected to a pressure transducer (–50, + 300 mmHg) and amplifier (Emka Technologies, Paris, France) to measure arterial blood pressure, and the venous catheter served to administer anesthetic supplements. To study the effect of ischemic hypoxia on blood pressure, SBP, DBP, and time elapsed between two successive R-waves of the QRS signal on the electrocardiogram—RR intervals—were recorded in spontaneously breathing anesthetized rats submitted to bilateral occlusion of the common carotid artery (OCC) for either 5 or 1 5 s. MBP was calculated using the values of SBP and DBP by the Iox 2.9.5.73 software (Emka Technologies, Paris, France) using the following mathematical formula: MAP = [SBP + 2 (DBP)]/3.

### Assessment of Blood Pressure and Heart Rate Regulation in Response to Hypoxia After Carotid Sinus Nerve Bilateral Denervation

To test the influence of CSN on blood pressure responses to hypoxia, rats were anesthetized with sodium pentobarbital (60 mg kg-1 i.p.), the carotid artery bifurcations were located, bilaterally, and CSNs were identified and sectioned. The femoral artery and vein were catheterized to measure arterial blood pressure and to administer anesthetic supplements, respectively. SBP, DBP, HR, and time elapsed between two successive R-waves of the QRS signal on the electrocardiogram—RR intervals—were continuously recorded during ischemic hypoxia induced by bilateral OCC during either 5 or 15 s. Mean RR intervals were plotted vs. SBP, DBP, and MBP. One animal implanted with a telemetry blood pressure transducer, as described above, was anesthetized and tilted at an angle of 75°, and blood pressure changes were acquired using the Data Acquisition System from Data Sciences International (DSI, St. Paul, MN, United States) before and after acute CSN resection.

### Assessment of Blood Pressure and Heart Rate Regulation in Response to Kilohertz Frequency Alternate Current Modulation of the Carotid Sinus Nerve

To evaluate the impact of acute Kilohertz frequency alternate current (KHFAC) modulation of the CSN on blood pressure and hypoxic responses, animals were implanted with bipolar sling cuff electrodes (90% platinum and 10% iridium, 100 μm inner diameter × 1 mm length, electrode surface area 0.4 × 0.5 mm^2^, 0.45 mm interelectrode center-to-center distance, CorTec, Freiburg, Germany). Fibrin glue (Tisseel, Baxter Healthcare, Compton, Newbury, United Kingdom) was used to secure the cuff to the CSN and to prevent current spread from the ends of the cuff. The effect of KHFAC modulation (blocking and stimulation) on respiratory rate, HR, blood pressure, and cardiorespiratory responses evoked by hypoxia was evaluated by applying to the cuff electrodes bilaterally as rectangular pulses a current of 2 mA for 20 Hz (CSN stimulation) and 50 kHz (blocking) as previously described (Sacramento et al., 2018). KHFAC was applied using a commercial current source (Keithley 6221, Tektronix, Bracknell, United Kingdom) for 10 s for CSN stimulation and 1 min for CSN blocking. To ensure near-equal current split, the cuff electrode impedances were measured in saline (154 mmol/l NaCl) before implantation, and the cuffs were matched for each animal based on < 10% difference in their impedance values. The current values are reported as peak-to-peak for each cuff, assuming an equal 50/50 split from the current source output. The impact of CSN blocking on cardiorespiratory responses to hypoxia was tested by delivering hypoxia (10% O_2_ balanced N_2_) through a mask to the animal for 1 min. Respiratory frequency and HR were recorded to determine the efficacy of KHFAC modulation of the CSN. Respiratory and cardiac variables were measured utilizing intercostal platinum wires for electromyography (EMG) and ECG placed subcutaneously across the diaphragm as previously described (Sacramento et al., 2018). EMG and ECG data were differentially recorded using Digidata Low Noise Data Acquisition System (Molecular Devices, Wokingham, United Kingdom). Blood pressure was measured through a catheter placed at the femoral artery and connected to a pressure transducer (–50, + 300 mmHg) and amplifier (Emka Technologies, Paris, France). At the end of experiments, animals were euthanized by an intracardiac overdose of pentobarbital. Death was confirmed by cervical dislocation.

### Evaluation of Cardiac Autonomic Nervous System

Analysis of heart rate variability (HRV) is a non-invasive indirect method to assess the cardiac sympatho-vagal balance and herein was used both in freely moving and anesthetized rats to evaluate autonomic neural regulation of heart rate. Power spectral analysis of HRV was performed to evaluate the balance between the sympathetic and parasympathetic components of the cardiac autonomic nervous system (Thireau et al., 2008; Fonseca-Pinto, 2011; Silva et al., 2017). In anesthetized animals, the femoral artery was cannulated under a dissection microscope, and the animals were transferred to a heating pad to maintain body temperature at 37.5 ± 0.5°C, thus avoiding cold stress sympathetic activation. The catheter was connected to a pressure transducer and amplifier to acquire MAP (model 603, HSE-HA GmgH, Harvard Apparatus, Madrid, Spain). HR was derived from the mean arterial pressure (MAP) curve obtained by HSE-Harvard Pulmodyn W software with an acquisition frequency of 500 Hz. The tachogram containing the RR signal was obtained after the identification of the peak of MAP in each cardiac cycle. In conscious animals, blood pressure was assessed by a radiotelemetry device placed in the abdomen. Animals were gentled for 10 min daily for 1 week prior to surgery to minimize any discomfort related to the experimental manipulation and to reduce data variability from indwelling blood pressure telemeter instrumentation. After transmitter implantation surgery, the animals were allowed to recover for 10 days before any measurements were recorded. HR and RR intervals were obtained using Iox 2.9.5.73 software (Emka Technologies, Paris, France) with an acquisition frequency of 500 Hz. Blood pressure recordings to calculate LF/HF index were selected using 300 s of stable recording before and after the interventions except for the KHFAC neuromodulation experimental setting, in which the intervals chosen were of 30 s, corresponding to the neurostimulation period chosen to avoid central hypoxia.

The RR plots obtained in both groups were interpolated at 10 Hz (a frequency suitable to catch all oscillations from heart rhythm in rats) using cubic splines. The algorithm used to obtain spectral non-parametric HRV indices was created in Matlab Software (MATLAB version 7.10.0. Natick, Massachusetts, United States: The MathWorks Inc.), using a fast fourier transform approach (Thireau et al., 2008) by Welch spectral estimation considering a 256-point window and 50% overlapped. Beyond the relative power obtained by the area under the spectral curve associated with slow (High Frequencies–Hf) and Fast (Low Frequencies-Lf) oscillations, the sympathovagal balance was also calculated using the ratio between Lf and Hf. Hf power represents the vagal control of the heart, modulated by breathing, whereas Lf power (more precisely, its normalized version) reflects primarily the sympathetic modulation of heart rate (Thireau et al., 2008). Frequencies are presented in normalized units, and graphs were obtained using Kubios Software (Thireau et al., 2008). In rodents, the lack of a standard protocol has been conducted in recent years to a set of studies regarding methodological issues (Silva et al., 2017). Concerning the particular issue of defining the frequency bands associated with Lf and Hf frequency ranges, data obtained in the 90’s suggest different frequency bands for rats (with limits from 0.195–0.6 Hz (Lf band) to 0.6–2.5 Hz (Hf band) and a ratio between Lf and Hf bands of 0.32 and from 0.02 to 0.195 Hz (Lf band) to 0.195 to 0.6 Hz (Hf band) and a ratio of 3–5 (Aubert et al., 1999); however, based in more recent findings in rodents, it is suggested the use of Lf in (0.15–1.5 Hz) and Hf in (1.5–4 Hz) as a good compromise to gauge the sympathetic and parasympathetic components of HRV (Thireau et al., 2008; Silva et al., 2017). These frequency ranges were used in this work.

### Statistical Analysis

Statistical analyses were performed using Prism version 8 (GraphPad Software Inc., La Jolla, CA, United States). Data are presented as mean ± SEM. Shapiro–Wilk normality tests were performed. The significance of the differences between the mean values was calculated by two-tailed Student’s *t*-test and two-way ANOVA with Bonferroni multiple comparison tests. Differences were considered significant at *p* < 0.05.

## Results

### Evaluation of Blood Pressure and Heart Rate Responses to Acute Hypoxic Hypoxia and Ischemic Hypoxia in Conscious and Anesthetized Animals

**FIGURE 1:**
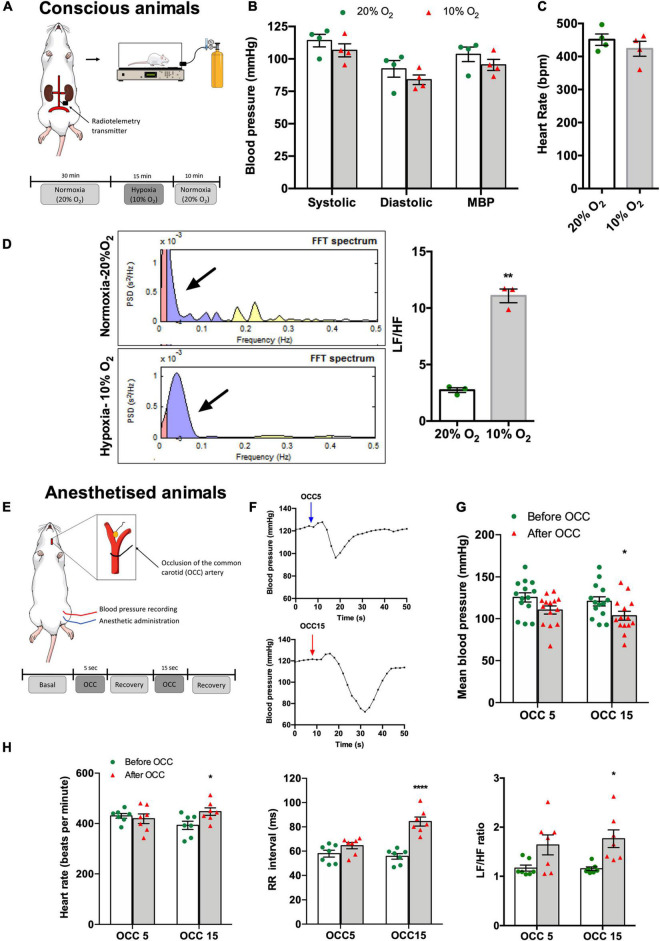
Effect of hypoxic and ischemic hypoxia on blood pressure, HR, and autonomic nervous system activity in conscious **(A–D)** and anesthetized animals **(E–H)**. **(A)** Schematic representation of the experimental protocol used to assess the effect of hypoxic hypoxia on physiological variables recorded in four conscious animals. Animals implanted with telemetry devices for blood pressure recording were submitted to 15 min of 10% O_2_ (balanced N_2_) after 30 min of baseline recording at normoxia (20% O_2_ balanced N_2_), *n* = 4. **(B)** Effect of hypoxic hypoxia on SBP, DBP, and MBP when compared with normoxia. **(C)** Effect of hypoxic hypoxia on HR (*n* = 4). **(D)** Effect of 15 min exposure to hypoxic hypoxia on autonomic function assessed by spectral analysis of HR. The left panel shows control examples of power spectral density in normoxia, *n* = 4 (top), and hypoxia, *n* = 3 (bottom). Frequencies are presented in normalized units. The right panel shows mean values of autonomic function assessed by the ratio between the percentage of Lf that represents the sympathetic component of the autonomic nervous system and the percentage of Hf that represents the parasympathetic component of the autonomic nervous system. **(E)** Schematic representation of the experimental protocol used to assess the effect of ischemic hypoxia on anesthetized animals. **(F)** Typical blood pressure responses to ischemic hypoxia assessed as OCC of 5 (top) and 15 s (bottom) of intensities. **(G)** Effect of 5 and 15 s OCC on MBP (*n* = 14). **(H)** Effect of 5 and 15 s of ischemic hypoxia on HR (left panel), on the RR intervals (middle panel), and autonomic function assessed by the spectral analysis of the HR and expressed as the ratio between the Lf and Hf of the spectra (right panel) (*n* = 7). Bars represent mean ± SEM. Shapiro–Wilk normality tests were performed, and all groups passed normality test. Whenever two groups were compared, two-tailed Student’s *t*-test was executed **(C,D)**, and when more than two groups were compared, two-way ANOVA with Bonferroni’s multiple comparison test was performed **(B,G,H)**; *p < 0.05, **p < 0.01, ****p < 0.0001 comparing values before and after the OCC.

Hypoxic hypoxia (10% O_2_) during 15 min in conscious animals (Figure 1A) did not significantly change SBP, DBP, and MBP although a decreasing trend was observed (*normoxia*: SBP = 114.13 ± 4.85 mmHg, DBP = 92.31 ± 6.41 mmHg, MBP = 103.51 ± 5.57 mmHg; *hypoxia:* SBP = 106.60 ± 5.01 mmHg, DBP = 83.92 ± 3.68 mmHg, MBP = 95.37 ± 4.25 mmHg, *n* = 4) (Figure 1B). In the same line, hypoxic hypoxia did not modify HR (*n* = 4; Figure 1C). In contrast, hypoxia increased the Lf band in the power spectrum of the HR variability (Figure 1D, left panel) as shown by a 305% increase in the Lf/Hf cardiac index (Lf/Hf normoxia = 2.73 ± 0.21, *n* = 4; Lf/Hf hypoxia = 11.08 ± 0.60, *n* = 3, Figure 1D, right panel), meaning that acute hypoxia promoted an increase in cardiac SNS activity.

Figures 1E–H show the effect of ischemic hypoxia assessed by OCC during 5 and 15 s on blood pressure, HR, and cardiac autonomic function in anesthetized animals (*n* = 7). Ischemic hypoxia produced a transient increase in MBP followed by a hypotensive effect as shown in the typical recordings presented in Figure 1F. Whereas the hypotensive effect produced by the OCC5 was non-significant (MBP: before OCC5 = 125.61 ± 5.50 mmHg, OCC5 = 110.56 ± 4.87 mmHg, *n* = 7), OCC15 significantly decreased MBP by 14.27% (MBP: before OCC15 = 120.88 ± 5.46 mmHg, OCC15 = 103.65 ± 5.46 mmHg, *n* = 7) (Figure 1G). In agreement, OCC5 did not change HR or RR intervals although producing a non-significant increase in Lf/Hf cardiac index of 21.50%, whereas OCC15 produced a significant increase of 13.83% in HR, of 34.04% in RR intervals, and of 53.00% in the Lf/Hf cardiac index (Figure 1H).

### Assessment of Blood Pressure and Heart Rate Regulation in Response to Hypoxia After Carotid Sinus Nerve Bilateral Denervation

**FIGURE 2:**
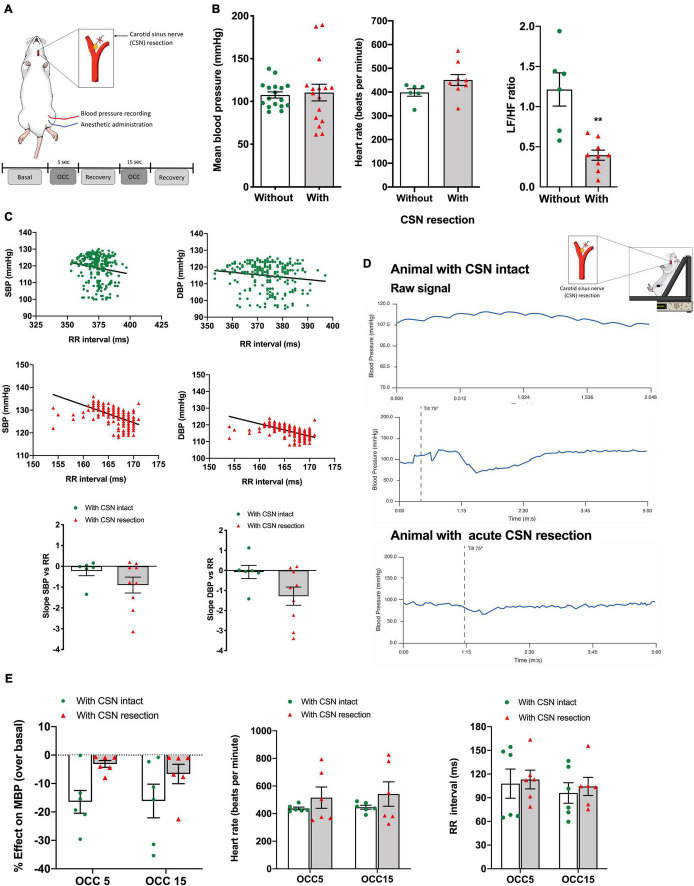
Effect of CSN acute resection on blood pressure, HR, autonomic function, baroreflex sensitivity, and blood pressure responses in basal conditions and in response to ischemic hypoxia in anesthetized animals. **(A)** Schematic representation of the protocol used to assess the impact of CSN on the physiological variables evaluated in basal conditions (normoxic atmosphere) and in response to ischemic hypoxia assessed as OCC of 5 and 15 s, **(B)** respectively, from left to right, the effect of bilateral CSN denervation on MBP (left panel), HR (middle panel), and autonomic function (right panel) assessed by the spectral analysis of the HR and expressed as the ratio between the Lf and Hf of the spectra in anesthetized rats (control; *n* = 17; CSN denervated, *n* = 16). **(C)** Top graphs show representative SBP and DBP correlations with the RR intervals in rats with CSN intact (green, *n* = 6) and with CSN resection (red, *n* = 9) obtained in the experimental setting shown in **(A)**. Graphs on the bottom show the mean values of the slope of the correlations between SBP and DBP and RR intervals. **(D)** Typical response to a tilt test in conscious animals implanted with telemetry devices for blood pressure recording. Top panel shows raw signal of MBP in a rat with CSN intact. Middle and bottom panels show, respectively, a tilt test performed in a rat with CSN intact and with bilateral resection of the CSN. **(E)** Effect of CSN denervation on MBP (left panel), HR (middle panel), and RR intervals (right panel in response to ischemic hypoxia of 5 and 15 s of intensity, *n* = 6). Bars represent mean ± SEM. Shapiro–Wilk normality tests were performed, and all groups passed normality test. Two-tailed Student’s *t*-test was accomplished **(B,C)** when comparing values with and without CSN denervation. When more than two groups were compared, two-way ANOVA with Bonferroni multicomparison test was performed **(E)**; **p < 0.01.

Figure 2 depicts the effect of acute CSN resection (Figure 2A) on blood pressure and HR regulation in both baseline and response to ischemic hypoxia as well as the result of a single experiment on the effect of CSN resection on the baroreflex. Acute CSN bilateral resection did not modify either MBP (Figure 2B, MBP without CSN resection = 107.50 ± 3.65 mmHg, *n* = 17; MBP with CSN resection = 110.3 ± 9.71 mmHg, *n* = 16) or HR (HR without CSN resection = 398.1 ± 15.75 beats per min, *n* = 17; HR with CSN resection = 450.6 ± 22.92, *n* = 16) but decreased sympathetic activation as shown by a significant decrease in Lf/Hf cardiac index of 67.19% (Figure 2B). Top panels of Figure 2C shows representative correlations between SBP and DBP and the RR intervals in animals with the CSN intact (green) and animals with bilateral resection of the CSN (Figure 2C). CSN resection showed a tendency, although non-statistically significant, to alter the slope of the correlation between SBP and DBP and the RR intervals [slope SBP vs. RR without CSN resection = −0.22 ± 0.22, *n* = 6; slope SBP vs. RR with CSN resection = −0.90 ± 0.30, *n* = 9 (*p* = 0.206); slope DBP vs. RR without CSN resection = −0.07 ± 0.33, *n* = 6; slope DBP vs. RR with CSN resection = −1.28 ± 0.45, *n* = 9 (*p* = 0.0748)] suggesting that CSN denervated animals exhibit significantly altered baroreflex sensitivity. To clarify these findings, baroreflex sensitivity was evaluated by measuring blood pressure and RR interval variation during a postural challenge, the tilt test (Figure 2D). Figure 2D shows typical recordings of blood pressure in anesthetized rats with (bottom panel) and without (middle panel) CSN resection, submitted to a tilt test, in which the animal was suspended at an angle of 75° (middle panel). Note that CSN resection alters the variations in MBP produced by tilting the animal, suggesting altered baroreflex sensitivity caused by denervation of the CSN. The effect of CSN resection was tested also in the MBP, HR, and RR intervals in response to ischemic hypoxia. Acute CSN denervation attenuated the decrease in MBP produced by ischemic hypoxia of 5 and 15 s (Figure 2E, left panel) in a non-significant manner (*n* = 6) without altering either HR or RR intervals (Figure 2E, middle and right panels).

### Assessment of Blood Pressure and Heart Rate Regulation in Response to Kilohertz Frequency Alternate Current Modulation of the Carotid Sinus Nerve

**FIGURE 3:**
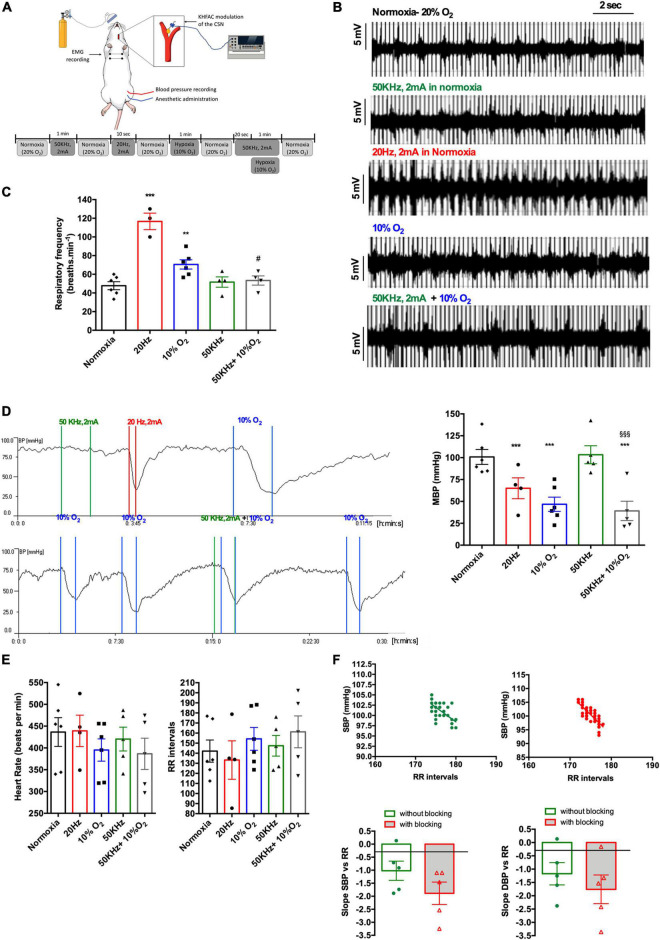
Effect of acute CSN electrical modulation on baseline cardiorespiratory variables and the cardiorespiratory response to hypoxic hypoxia in anesthetized rats. **(A)** Schematic representation of the experimental setting and protocol used to evaluate the effect of electrical blocking of the CSN on the physiological variables; **(B)** panel shows, respectively, from top to bottom, EMG representative recordings of animals in normoxia (*n* = 6; 20% O_2_ balanced N_2_), under electrical blocking (*n* = 6; 50 kHz, 2 mA, 1 min), under electrical stimulation (*n* = 6, 20 Hz, 2 mA, 10 s), in response to hypoxia (*n* = 6, 10% O_2_ balanced N_2_, 1 min), and under electrical block plus hypoxia (*n* = 6; 50 kHz, 2 mA plus 10% O_2_). **(C)** Shows the effect of CSN neuromodulation–stimulation (20 Hz, 2 mA, 10 s, *n* = 4) and blocking (50 kHz, 2 mA, 1 min, *n* = 4) on baseline respiratory frequency and respiratory frequency evoked by hypoxic hypoxia (10% O_2_ balanced N_2_, 1 min, *n* = 6) expressed as bpm. **(D)** Left panel shows a typical recording of the effect of electrical blocking (50 kHz, 2 mA, 1 min) and stimulation (20 Hz, 2 mA, 10 s) of the CSN and the effect of hypoxic hypoxia (10% O_2_ balanced N_2_, 1 min) on MBP measured at the femoral artery. Right panel show the mean values for the effect of electrical blocking on MBP in normoxia and hypoxia. Note that electrical blocking did not modify MBP and that CSN stimulation elicited a hypotensive response such as the produced by hypoxic hypoxia. The panel at the bottom shows the effect of blocking (50 kHz, 2 mA, 1 min) on MBP response to hypoxic hypoxia (10% O_2_ balanced N_2_, 1 min). Note that electrical blocking of CSN does not seem to affect blood pressure response to hypoxic hypoxia. CSN electrical modulation was applied bilaterally to the CSN through electrode cuffs. Stimuli were applied between colored lines, and colors represent, respectively, green—electrical blocking (50 kHz, 2 mA, 1 min), red—electrical stimulation (20 KHz, 2 mA, 10 s), and blue—hypoxic hypoxia (10% O_2_ balanced N_2_, 1 min). To test CSN electrical blocking plus hypoxia, the current was initiated 30 s before submitting the animals to hypoxia. **(E)** Effect of CSN neuromodulation–stimulation (20 Hz, 2 mA, 10 s, *n* = 4) and blocking (50 kHz, 2 mA, 1 min, *n* = 4) and hypoxia (*n* = 6) on HR and RR intervals. **(F)** Top panel shows correlations between SBP and RR intervals in animals without CSN electrical blocking (green, *n* = 6) and when submitted to electrical blocking (red, *n* = 6). Correlations were performed with values obtained during 1 min at a sampling rate of 1,000 Hz. Bottom panels show the impact of electrical neuromodulation of the CSN on the mean values of the slope of the correlations between SBP and DBP and RR intervals. Bars represent mean ± SEM. Shapiro–Wilk normality tests were performed, and all groups passed normality test. One-way ANOVA with Bonferroni’s multiple comparisons test was executed in series depicted in **(C–E)**; **p < 0.01, ***p < 0.001 vs. normoxia; ^#^*p* < 0.05, ^$$$^*p* < 0.001 vs. hypoxia. Whenever two groups were compared a two-tailed Student’s *t*-test was performed **(F)**.

Figure 3 shows the effect of CSN electrical neuromodulation on respiratory frequency, blood pressure, and HR in normoxic conditions and response to hypoxic hypoxia in anesthetized animals (Figure 3A). Typical EMG recordings of respiratory frequency in response to hypoxic hypoxia, CSN stimulation, and CSN high frequency blocking are shown in Figure 3B. As expected, 1 min hypoxic hypoxia (10% O_2_) significantly increased respiratory frequency by 47.66% (*normoxia* = 47.78 ± 4.28 bpm; *hypoxia* = 70.56 ± 4.98 bpm; *n* = 6) (Figure 3C). Electrical stimulation of the CSN significantly increased respiratory frequency, confirming that the electrodes were correctly placed (respiratory frequency 20 Hz = 116.70 ± 8.82 bpm, *n* = 6). As previously described (Sacramento et al., 2018) and as shown in Figure 3C, high frequency blocking of the CSN (50 kHz, 2 mA) did not modify respiratory frequency in normoxia (respiratory frequency 50 kHz = 51.67 ± 5.53 bpm, *n* = 6) but abolished the ventilatory hypoxic response (respiratory frequency 50 kHz + 10% O_2_ = 53.33 ± 4.91 bpm, *n* = 6). Hypoxic hypoxia applied for 1 min decreased MBP by 53.6% (normoxic MBP = 100.8 ± 8.56 mmHg: hypoxia MBP = 46.77 ± 8.15 mmHg, *n* = 6, Figure 3D). Likewise, stimulation of CSN with 20 Hz frequency produced a decrease in blood pressure of 24.1% (MBP 20 Hz = 65.0 ± 11.92 mmHg) (Figure 3D). Similarly, to the effect of high frequency blocking on respiratory frequency, electrical blocking of the CSN blocking did not modify MBP in normoxia (MBP 50 KHz = 103.3 ± 10.29 mmHg, *n* = 6, Figure 3D). In contrast, it did not produce any alteration in MBP in response to hypoxic hypoxia (MBP 50 KHz + 10% O_2_ = 39.14 ± 11.09, Figure 3D), indicating a possible decoupling of CB-mediated ventilatory and blood pressure responses to hypoxia. Note also that the high frequency blocking of the CSN did not modify both HR and the RR intervals (Figure 3E). The effect of electrical blocking was also tested on the correlations between SBP and DBP and the RR intervals (Figure 3F). The top panel of Figure 3F shows correlations between SBP and RR intervals in CSN without electrical stimulation (green) and when the animals were submitted to electrical blocking for 1 min (red) (Figure 3F, top panel). High frequency electrical blocking of the CSN did not change the correlation between SBP and DBP and the RR intervals [slope SBP vs. RR without CSN blocking = −1.02 ± 0.37; slope SBP vs. RR with CSN electrical blocking = −1.89 ± 0.43 (*n* = 6, *p* = 0.17); slope DBP vs. RR without CSN electrical blocking = −1.173 ± 0.342; slope DBP vs. RR with CSN resection = −1.759 ± 0.54 (*n* = 6, *p* = 0.42)].

## Discussion

This study assessed the impact of the CSN in systemic blood pressure under normoxic and hypoxic conditions, aiming for a better understanding of the crosstalk between the chemoreceptors and blood pressure control systems. Our study confirms that functional ablation of the CSN, either surgically or through electrical neuromodulation, does not significantly influence baseline hemodynamic parameters nor blood pressure modulation by hypoxic/ischemic challenges despite a decreased sympathetic tone induced by loss of CSN function. We hypothesize that bilateral denervation/electrical blockade of the CSN and subsequent abolition of CB afferent chemoreflex and carotid-sinus baroreflex are compensated by redundant mechanisms that respond to acute changes in blood gases, independently of the autonomic resetting that occurs upon hindrance of CSN transmission.

It was previously shown that that baseline values of respiratory and cardiovascular parameters do not differ before and after unilateral chemoreceptor deactivation through ligation and sectioning of the left CB artery for Katayama et al. (2019).

The same authors observed that unilateral electrical stimulation of the carotid sinus and the CSN activates both the carotid baroreflex and chemoreflex as previously described (Gonzalez et al., 1994; Marshall, 1994, 1998), resulting in hypotension in conscious animals (Katayama et al., 2015). Additionally, it was also demonstrated that the hypotensive response after electrical stimulation of the carotid sinus was enhanced by carotid chemoreceptor deactivation (Katayama et al., 2015), suggesting that an intact bilateral chemoreflex counteracts the hypotensive effects carotid sinus stimulation.

Herein, we performed bilateral surgical or electrical ablation of the CSN, thus preventing complete conveying of the information from carotid chemo and baroreceptors to the central nervous system, and we observed no changes in blood pressure or HR despite a significant decrease in cardiac sympathetic activity in both normoxic and hypoxic conditions.

Looking at the correlation between the SBP and DBP and RR intervals, we observed no significant differences between the slopes, suggesting that CSN resection does not affect the feedback loop among blood pressure and HR in basal conditions. Pijacka et al. (2018) report that CSN denervation resulted in abolishment of the baroreceptor reflex, which was not perceivable after selective CB resection. The single experiment we performed in a CSN-resected animal submitted to a head-up tilt test showed a less pronounced drop in blood pressure when comparing the pre-denervation period with the post-denervation period, indicating that despite causing no significant changes in systemic blood pressure control, CSN-denervated animals may present an impaired hemodynamic response to head-up tilting, also observed by Pijacka et al. (2018).

Immediate cardiovascular responses to acute hypoxia are known to be species-dependent, primarily mediated by the aortic bodies with a smaller contribution of the CB and followed by hypoxic vasodilatory response controlled by local autoregulatory mechanisms at peripheral vascular beds (Marshall, 1998; Rohlicek et al., 2002; Cowburn et al., 2017). In humans, CBs play a critical role in adapting ventilation and maintaining arterial pressure during hypoxia, whereas the HR response is mainly mediated by aortic bodies (Niewinski et al., 2014; Pijacka et al., 2018). In anesthetized rats, hypoxic hypoxia (10% O_2_ in N_2_), which stimulates both CBs and aortic bodies, and carbon monoxide hypoxia (30% O_2_ in N_2_ with CO addition) that stimulates only the aortic bodies, induced an increase in cardiac output, cardiac contractility, SBP/DBP, aortic blood pressure, total peripheral resistance, and pulmonary arterial pressure (Fitzgerald et al., 2013) with the CBs having a higher effect in the majority of these variables except for blood pressure—whose major control seems to be assured by the aortic bodies (Fitzgerald et al., 2013). In contrast, sustained or chronic hypoxia leads to distinct ventilatory and hemodynamic responses that are not easily attributed to chemoreflex or baroreflex independently and involve resetting of the feedback loops (Niewinski et al., 2014; Lohmeier et al., 2016; Pijacka et al., 2018). However, the relation of the CBs with sympathetic tone is irrefutable in either acute conditions or response to chronic stimuli (Prabhakar et al., 2005; McBryde et al., 2017; Sacramento et al., 2020) with a clear involvement of these organs in long-term increase in sympathetic vasoconstrictor outflow in several disease states (Conde et al., 2014; Pijacka et al., 2018).

Herein, we observed no significant effects of acute hypoxic hypoxia in baseline hemodynamic parameters in conscious animals implanted with continuous blood pressure monitoring telemeters although there was a significant activation of the SNS as assessed by HR variability analysis. In a setting of ischemic hypoxia, we also observed no changes in blood pressure although there was an increase in sympathetic activation, significant only for a 15-s ischemia period.

To assess the role of the CB chemoreceptors in acute blood pressure control in response to hypoxia, we surgically denervated the CSN and evaluated acute changes in BP, sympathetic activity in hypoxic hypoxia conditions. We observed that blood pressure was not significantly modified in acutely CSN denervated animals, but the increase in SNS activity in response to ischemic hypoxia was lost in animals submitted to CSN denervation. Acute CSN resection also diminished the hypotensive effect evoked by hypoxia. In these animals, the decrease in BP in response to hypoxia was lower than in animals with intact CSN. To confirm these findings, we functionally abolished CSN activity by KHFAC (50 kHz, 2 mA) and observed that neither baseline blood pressure nor the adaptive blood pressure response to hypoxic hypoxia was affected by the electrical blockade. Our results support that functional or surgical blockade of the CSN does not impact basal blood pressure or basal ventilation in contrast to electrical stimulation of the CSN that mimics the ventilatory and hemodynamic responses evoked by hypoxic and ischemic hypoxia.

We concluded that, in acute settings, the chemoreflex and baroreflex control of blood pressure are dissociated in terms of hypoxic response but integrated to generate an effector response to a given change in arterial pressure. Chemoreceptor activation caused by acute hypoxic or ischemic hypoxia increased sympathetic activity in both conscious and anesthetized animals. However, baseline blood pressure was not affected by acute hypoxia except for sustained hypoxic challenges, namely, OCC for 15 s, indicating that hemodynamic compensatory regional mechanisms triggered by acute hypoxia are effective in maintaining blood pressure for short-term stimuli but not for longer challenges. Paton’s group recently reported that CSN sparing selective ablation of the CBs assures functional integrity of the carotid sinus baroreceptors in spontaneously hypertensive rats, demonstrating the importance of CBs in the hemodynamic response to hypoxia and hypercapnia in hypertension (Pijacka et al., 2018). Regarding the contribution of the CB to the development and maintenance of hypertension, our work agrees with previous results obtained by other groups in which it was observed that carotid sinus denervation prevented arterial pressure from reaching hypertensive levels and decreased sympathetic activity in spontaneous hypertensive young rats (Pijacka et al., 2018).

To conclude, chemoreflex and baroreflex short-term regulation of blood pressure are dissociated in terms of hypoxic response but integrated to generate an effector response to a given change in arterial pressure. Based on our findings, we postulate that the clinical use of Hf stimulation to modulate CSN activity is devoid of hypoxic-induced pressure fluctuations.

## Data Availability Statement

The raw data supporting the conclusions of this article will be made available by the authors upon request, without undue reservation.

## Ethics Statement

The animal study was reviewed and approved by the Animal Welfare Body of Faculdade de Ciências Médicas | Nova Medical School and by the Direcção Geral de Veterenária (DGAV), Portugal.

## Author Contributions

SC, MG, and MR-O designed the experiments. SC, JS, BM, and MG performed the experiments. SC, JS, BM, MG, RF-P, and MR-O analyzed the data. SC, BM, RF-P, and MG contributed to manuscript preparation. SC and MG wrote the manuscript. All authors reviewed and accepted the manuscript.

## Conflict of Interest

Part of this study, in particular Figures 1, 3 were funded by GlaxoSmithKline Bioelectronics R&D unit. The funder had the following involvement with the study: decision to publish. The authors declare that the research was conducted in the absence of any commercial or financial relationships that could be construed as a potential conflict of interest.

## Publisher’s Note

All claims expressed in this article are solely those of the authors and do not necessarily represent those of their affiliated organizations, or those of the publisher, the editors and the reviewers. Any product that may be evaluated in this article, or claim that may be made by its manufacturer, is not guaranteed or endorsed by the publisher.
